# Cardiovascular risk in patients with and without diabetes presenting with chronic coronary syndrome in 2004–2016

**DOI:** 10.1186/s12872-021-02312-y

**Published:** 2021-12-04

**Authors:** Esben Skov Jensen, Kevin Kris Warnakula Olesen, Christine Gyldenkerne, Pernille Gro Thrane, Lisette Okkels Jensen, Bent Raungaard, Per Løgstrup Poulsen, Reimar Wernich Thomsen, Michael Maeng

**Affiliations:** 1 Department of Cardiology, Aarhus University Hospital, Palle Juul-Jensens Boulevard 99, Aarhus N, Denmark; 2 Department of Cardiology, Odense University Hospital, Odense, Denmark; 3 Department of Cardiology, Aalborg University Hospital, Aalborg, Denmark; 4 Steno Diabetes Center Aarhus, Aarhus University Hospital, Aarhus, Denmark; 5 Department of Clinical Epidemiology, Aarhus University Hospital, Aarhus, Denmark

**Keywords:** Diabetes, Coronary artery disease, Major adverse cardiovascular event, Trend

## Abstract

**Background:**

It was recently shown that new-onset diabetes patients without previous cardiovascular disease have experienced a markedly reduced risk of adverse cardiovascular events from 1996 to 2011. However, it remains unknown if similar improvements are present following the diagnosis of chronic coronary syndrome. The purpose of this study was to examine the change in cardiovascular risk among diabetes patients with chronic coronary syndrome from 2004 to 2016.

**Methods:**

We included patients with documentation of coronary artery disease by coronary angiography between 2004 and 2016 in Western Denmark. Patients were stratified by year of index coronary angiography (2004–2006, 2007–2009, 2010–2012, and 2013–2016) and followed for two years. The main outcome was major adverse cardiovascular events (MACE) defined as myocardial infarction, ischemic stroke, or death. Analyses were performed separately in patients with and without diabetes. We estimated two-year risk of each outcome and adjusted incidence rate ratios (aIRR) using patients examined in 2004-2006 as reference.

**Results:**

Among 5931 patients with diabetes, two-year MACE risks were 8.4% in 2004–2006, 8.5% in 2007–2009, and then decreased to 6.2% in 2010–2012 and 6.7% in 2013–2016 (2013–2016 vs 2004–2006: aIRR 0.70, 95% CI 0.53–0.93). In comparison, 23,540 patients without diabetes had event rates of 6.3%, 5.2%, 4.2%, and 3.9% for the study intervals (2013–2016 vs 2004–2006: aIRR 0.57, 95% CI 0.48–0.68).

**Conclusions:**

Between 2004 and 2016, the two-year relative risk of MACE decreased by 30% in patients with diabetes and chronic coronary syndrome, but slightly larger absolute and relative reductions were observed in patients without diabetes.

**Supplementary Information:**

The online version contains supplementary material available at 10.1186/s12872-021-02312-y.

## Background

Among patients with diabetes, randomized clinical studies have shown that multifactorial medical intervention with tight regulation of blood glucose, blood pressure, and lipid-levels reduces the risk of myocardial infarction (MI) and premature death [1]. This subsequently led to changes of the diabetes guidelines with focus on prophylactic multifactorial intervention [2–6]. We recently found substantial reduction in the risk of MI among new-onset type 2 diabetes patients in Denmark without previous cardiovascular disease, simultaneous with the implementation of multifactorial intervention [7]. Further, following documentation of coronary artery disease (CAD) in patients with diabetes, the management and treatment of CAD have also improved in the last decades with the documentation of coronary artery bypass grafting (CABG) being superior to percutaneous coronary intervention (PCI) when multivessel disease is present, the implementation of fractional flow reserve (FFR) measurement as an important diagnostic tool, and the development of newer-generation drug-eluting stents (DES) with lower risk of stent thrombosis being the most important improvements [8–10]. However, whether cardiovascular risk for diabetes patients with chronic coronary syndrome has changed over the last decades has not been examined in the setting of daily clinical practice on a nationwide level. Therefore, we investigated changes in cardiovascular risk among diabetes patients with chronic coronary syndrome from 2004 to 2016 and used patients without diabetes as a comparison cohort. We hypothesized that substantial improvements in cardiovascular risk had taken place.

## Methods

### Data sources

The Western Denmark Heart Registry is a clinical database that provides prospective registration of all patients in Western Denmark undergoing cardiac intervention such as coronary angiography (CAG), PCI, and CABG since 1999. The registry has previously been described in detail [11]. Using each patient’s unique 10-digit identifier, patients can be linked with other national health care registries, including the Danish National Prescription Registry, the Civil Registration System, the Danish Register of Causes of Death, and the Danish National Patient Registry [12–15].

### Patient selection

**Fig. 1:**
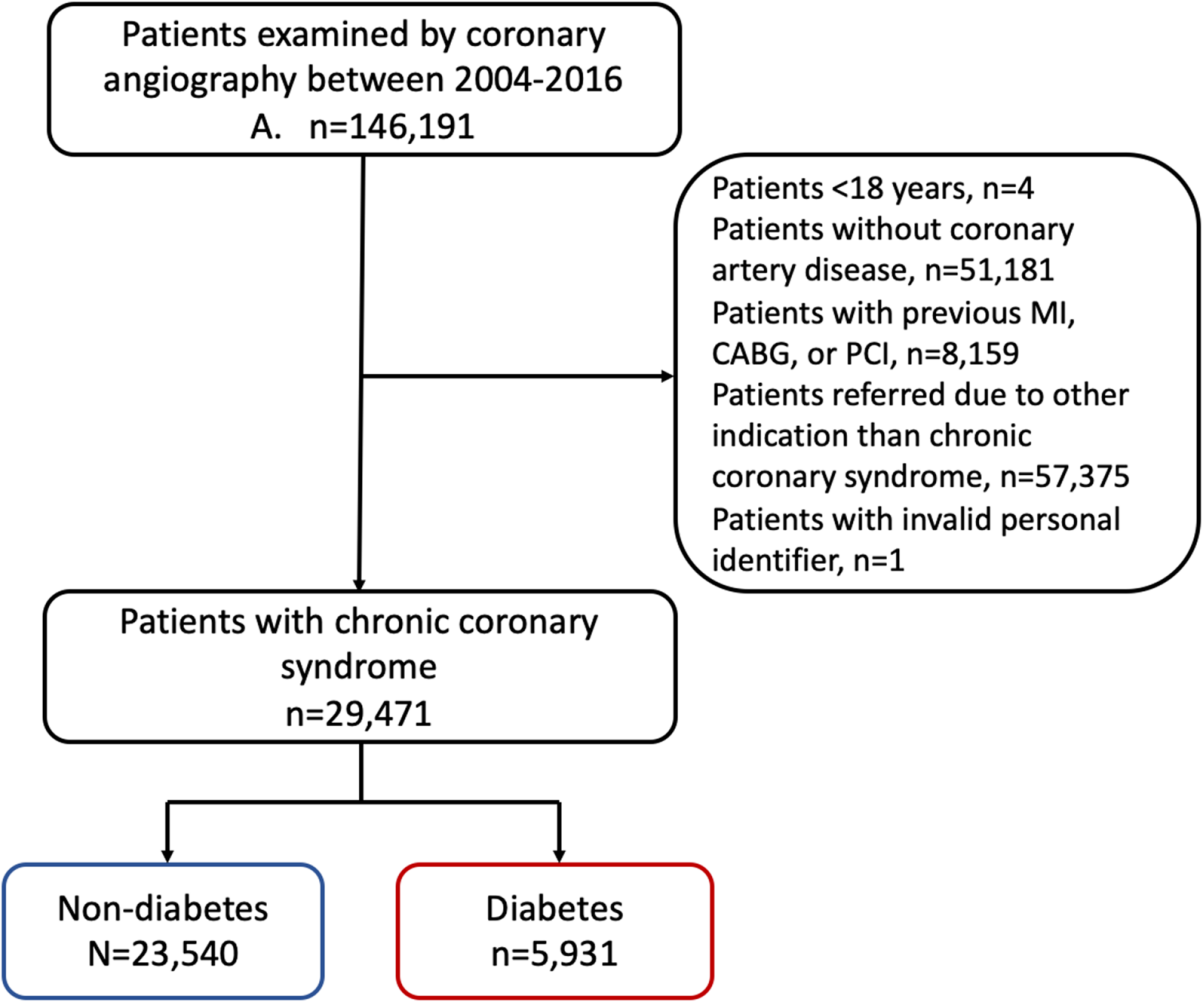
Selection of patients with newly diagnosed chronic coronary syndrome determined by CAG from January 1, 2004, to December 31, 2016, in Western Denmark

Patients undergoing CAG were identified using first-time procedures registered in the Western Denmark Heart Registry from 2004 through 2016 (n = 146,191) (Fig. 1). If a patient had multiple CAGs registered during this time, the first was considered the index examination. Four patients < 18 years and 51,181 patients with no CAD were excluded from this analysis. Since we aimed to assess risk following the first-time diagnosis of chronic coronary syndrome by CAG, we excluded 8159 patients with previous MI, PCI, or CABG. Patients referred for CAG due to a different indication than chronic coronary syndrome were also excluded (n = 57,375).

### CAD

Presence and extent of CAD were entered into the database by the interventional cardiologist immediately following examination. CAD was classified as either obstructive disease in 1, 2, or 3 vessels (with obstructive disease defined as > 50% diameter stenosis and FFR ≤ 0.80 if measured) or as diffuse CAD defined as non-significant CAD involving > 1 vessel. Patients with only a single stenosis < 50% or FFR > 0.80 if measured were classified as no CAD and excluded from the study.

### Diabetes

Diabetes was defined as either (1) diet treatment only, non-insulin anti-diabetic treatment, or insulin (± non-insulin anti-diabetic treatment) as registered in the Western Denmark Heart Registry, (2) diabetes diagnosis prior to CAG in the Danish National Patient Registry, or (3) collecting one or more prescriptions of insulin or non-insulin anti-diabetic treatment less than six months before CAG according to the Danish National Prescription Registry [12].

### Comorbidity

Comorbidities were ascertained through the Danish National Patient Registry relying on diagnoses prior to CAG with full look-back (from 1977 and onwards). Information regarding smoking status, body mass index (BMI), and hypertension was ascertained through the Western Danish Heart Registry. We estimated burden of comorbidity using a modified Charlson’s Comorbidity Index score, in which ‘Diabetes, type I and II’ and ‘Diabetes with end-organ failure’ were excluded in the final score [16].

### Medication

Records of treatment with aspirin, adenosine diphosphate (ADP) receptor inhibitor, angiotensin-converting enzyme (ACE) inhibitor/angiotensin II receptor blocker (ARB), beta-blocker, and statin were collected from the Danish National Prescription Database. Medical treatment prior to CAG was defined as one or more redeemed prescriptions six months or less before CAG. Changes in medical treatment because of the CAG or peri-procedural diagnosis were investigated by looking at redeemed prescriptions six months or less after CAG in patients who completed six months of follow-up (n = 29,071) (Additional file 1: Tables S1 and S2).

### Outcomes

The primary outcome was major adverse cardiovascular event (MACE); a composite of MI, ischemic stroke, and all-cause death. Secondary outcomes were the individual components of MACE, cardiac death, PCI, and CABG.

MI and ischemic stroke were identified in the Danish National Patient Registry [17, 18]. Vital status (alive, death, or emigration) was obtained through the Danish Civil Registration System [15]. Cardiac death included deaths resulting from ischemic heart disease, sudden cardiac death, heart failure, or sudden death, unspecified, according to death certificates from the Danish Register of Causes of Death [14].

Anatomical Therapeutic Chemical (ATC) codes used in the Danish Prescription Registry and International Classification of Diseases 10 (ICD-10) codes used in the Danish National Health Registry and the Danish Register of Causes of Death are listed in supplemental material of previous work [19].

### Statistical analysis

Patients with chronic coronary syndrome were stratified by diabetes status at the time of examination and year of index CAG (2004–2006, 2007–2009, 2010–2012, and 2013–2016). We estimated two-year risks (cumulative incidence proportions) of MACE, MI, ischemic stroke, all-cause death, cardiac death, PCI, and CABG. Follow-up continued until an outcome event, death, emigration, or 24 months after CAG. Cumulative incidence proportion curves were constructed. We estimated the incidence rate ratio (IRR) using a modified Poisson regression with a robust variance–covariance estimator using the natural log of person-years as the offset [20]. IRRs were adjusted for sex, age, hypertension, previous ischemic stroke, peripheral artery disease, smoking, statin treatment, antiplatelet treatment, and oral anticoagulant treatment. Analyses of MACE, ischemic stroke, cardiac death, and all-cause death were additionally adjusted for atrial fibrillation and heart failure [21]. Patients examined between 2004 and 2006 were used as reference group throughout analyses.

We performed a number of sensitivity analyses. First, two-year MACE risks were compared between patients with and without diabetes (Additional file 1: Table S3). Secondly, we conducted a subgroup analysis of patients diagnosed with obstructive CAD at index CAG (n = 23,858) (Additional file 1: Tables S4 and S5). Other analyses included stratifying by sex and age above or below 70 years (Additional file 1: Tables S6, S7, S8, and S9). Lastly, we performed an analysis of revascularization patterns as a consequence of the angiographic findings defined as PCI or CABG within three months after index CAG (Additional file 1: Table S10). Stata/MP 16.0 (StataCorp LLC, College Station, TX, USA) was used for all analyses.

## Results

A total of 29,471 patients with chronic coronary syndrome, of whom 5931 (20%) had diabetes, were included and eligible for analyses.

### Baseline characteristics

Patient characteristics are outlined in Table 1 (diabetes) and Table 2 (non-diabetes). In general, similar changes in baseline characteristics were observed for diabetes and non-diabetes patients. The median age increased from 67 to 69 years for patients with diabetes and from 66 to 68 years for patients without diabetes from 2004–2006 to 2013–2016. We observed a reduction in the proportion of active smokers. Median BMI was 29 for patients with diabetes and 27 for patients without diabetes throughout the study period. Comorbidities increased in both groups with increasing Charlson’s Comorbidity Index scores. The extent of CAD changed over time with a decrease in obstructive multivessel disease and increased presence of diffuse non-significant CAD.

**Table 1:** Baseline characteristics in patients with diabetes

	2004–2006 n = 1066		2007–2009 n = 1507		2010–2012 n = 1523		2013–2016 n = 1835	
Median age, years (IQR)	67 (59–73)		67 (60–74)		68 (61–75)		69 (61–75)	
Male sex	780	73.2	1061	70.4	1058	69.5	1301	70.9
Family history of ischemic heart disease	486	45.6	671	44.5	697	45.8	795	43.3
Active smoker	239	22.4	313	20.8	335	22.0	363	19.8
*Comorbidity*								
Hypertension	834	78.2	1272	84.4	1353	88.8	1583	86.3
Previous ischemic stroke	37	3.5	52	3.5	60	3.9	75	4.1
Atrial fibrillation	86	8.1	141	9.4	159	10.4	219	11.9
Peripheral artery disease	101	9.5	162	10.7	167	11.0	184	10.0
Heart failure	116	10.9	186	12.3	155	10.2	163	8.9
Renal disease	32	3.0	51	3.4	67	4.4	92	5.0
Mean eGFR, mL/min (IQR)	84 (65–101)		88 (68–107)		91 (71–108)		90 (71–108)	
Median BMI, kg/m^2^ (IQR)	29 (26–32)		29 (26–33)		29 (26–33)		29 (26–33)	
*Modified Charlson Comorbidity Index score*								
0 points	853	80.0	1111	73.7	1111	72.9	1286	70.1
1 point	152	14.3	275	18.2	252	16.5	298	16.2
2 point	58	5.4	105	7.0	144	9.5	207	11.3
≥ 3 points	3	0.3	16	1.1	16	1.1	44	2.4
*CAD extent*								
1 VD	307	28.8	450	29.9	502	33,0	570	31.1
2 VD	269	25.2	336	22.3	312	20.5	400	21.8
3 VD	438	41.1	456	30.3	348	22.8	393	21.4
Diffuse VD	52	4.9	265	17.6	361	23.7	472	25.7
**Medication**								
*Statin*								
Before	868	81.4	1244	82.5	1223	80.3	1441	78.5
After	972	92.7	1349	91.4	1358	90.4	1598	88.6
*Aspirin*								
Before	876	82.2	1200	79.6	1162	76.3	1279	69.7
After	941	89.7	1284	87.0	1285	85.5	1446	80.2
*ADP-inhibitor*								
Before	32	3.0	42	2.8	63	4.1	135	7.4
After	519	49.5	655	44.4	651	43.3	763	42.3
*Vitamin K antagonists*								
Before	91	8.5	126	8.4	131	8.6	144	7.8
After	123	11.7	159	10.8	151	10.0	181	10.0
*Non-vitamin K antagonists*								
Before	0	0.0	0	0.0	7	0.5	88	4.8
After	0	0.0	0	0.0	14	0.9	107	5.9
*Beta-blocker*								
Before	732	68.7	894	59.3	820	53.8	867	47.2
After	820	78.2	1050	71.1	1005	66.9	1084	60.1
*ACE inhibitor*								
Before	500	46.9	758	50.3	736	48.3	755	41.1
After	540	51.5	791	53.6	733	48.8	727	40.3
*ARB*								
Before	290	27.2	494	32.8	505	33.2	609	33.2
After	302	28.8	490	33.2	499	33.2	613	34.0
*Thiazides*								
Before	231	21.7	317	21.0	331	21.7	298	16.2
After	247	23.5	309	20.9	317	21.1	281	15.6
*Calcium channel blocker*								
Before	423	39.7	621	41.2	662	43.5	705	38.4
After	446	42.5	695	47.1	733	48.8	811	45.0
*Insulin*								
Before	349	32.7	495	32.8	503	33.0	547	29.8
After	377	35.9	538	36.4	524	34.9	572	31.7
*Non-insulin*								
Before	644	60.4	936	62.1	1042	68.4	1346	73.4
After	630	60.1	934	63.3	1035	68.9	1306	72.4

Values are numbers and percentages unless otherwise stated

*ACE* angiotensin converting enzyme, *ADP* adenosine diphosphate, *ARB* angiotensin-II receptor blocker, *BMI* body mass index, *CAD* coronary artery disease, *eGFR* estimated glomerular filtration rate, *IQR* inter-quartile range, *VD* vessel disease

**Table 2:** Baseline characteristics in patients without diabetes

	2004–2006 n = 4847		2007–2009 n = 6104		2010–2012 n = 5547		2013–2016 n = 7042	
Mean age, years (IQR)	66 (58–74)		67 (59–74)		67 (59–75)		68 (59–75)	
Male sex	3554	73.3	4279	70.1	3732	67.3	4843	68.8
Family history	2290	47.2	2892	47.4	2687	48.4	3218	45.7
Active smoker	1293	26.7	1401	23.0	1275	23.0	1467	20.8
*Comorbidity*								
Hypertension	2875	59.3	3949	64.7	3736	67.4	4488	63.7
Previous ischemic stroke	42	0.9	88	1.4	113	2.0	158	2.2
Atrial fibrillation	391	8.1	499	8.2	474	8.5	643	9.1
Peripheral artery disease	260	5.4	361	5.9	363	6.5	427	6.1
Heart failure	390	8.0	434	7.1	357	6.4	358	5.1
Renal disease	61	1.3	93	1.5	91	1.6	171	2.4
Mean eGFR, mL/min (IQR)	81 (66–96)		86 (70–102)		89 (74–104)		89 (74–104)	
Median BMI, kg/m^2^ (IQR)	27 (24–29)		27 (24–30)		27 (24–29)		27 (24–29)	
*Modified Charlson Comorbidity Index score*								
0 points	3931	81.1	4568	74.8	3917	70.6	4799	68.1
1 point	607	12.5	929	15.2	893	16.1	1162	16.5
2 point	222	4.6	411	6.7	488	8.8	696	9.9
≥ 3 points	87	1.8	196	3.2	249	4.5	385	5.5
*CAD extent*								
1 VD	1764	36.4	2321	38.0	2049	36.9	2661	37.8
2 VD	1236	25.5	1437	23.5	1141	20.6	1391	19.8
3 VD	1530	31.6	1386	22.7	1029	18.6	1132	16.1
Diffuse VD	317	6.5	960	15.7	1328	23.9	1858	26.4
**Medication**								
*Statin*								
Before	3141	64.8	4136	67.8	3667	66.1	4490	63.8
After	4347	91.4	5453	90.9	4879	89.1	6195	89.0
*Aspirin*								
Before	3795	78.3	4621	75.7	4009	72.3	4717	67.0
After	4105	86.3	5067	84.5	4555	83.2	5500	79.0
*ADP-inhibitor*								
Before	148	3.1	145	2.4	215	3.9	487	6.9
After	2540	53.4	2962	49.4	2535	46.3	3240	46.5
*Vitamin K antagonists*								
Before	354	7.3	427	7.0	360	6.5	376	5.3
After	519	10.7	535	8.9	471	8.6	484	7.0
*Non-vitamin K antagonists*								
Before	0	0.0	< 5	0.0	38	0.7	268	3.8
After	0	0.0	< 5	0.0	61	1.1	347	5.0
*Beta-blocker*								
Before	3348	69.1	3674	60.2	2868	51.7	2756	39.1
After	3582	75.3	4219	70.3	3498	63.9	3707	53.2
*ACE inhibitor*								
Before	1260	26.0	1643	26.9	1587	28.6	1590	22.6
After	1534	32.3	1947	32.5	1749	31.9	1694	24.3
*ARB*								
Before	667	13.8	976	16.0	978	17.6	1479	21.0
After	731	15.4	1041	17.4	1057	19.3	1564	22.5
*Thiazides*								
Before	891	18.4	1077	17.6	954	17.2	917	13.0
After	964	20.3	1111	18.5	930	17.0	914	13.1
*Calcium channel blocker*								
Before	1601	33.0	1958	32.1	1741	31.4	1982	28.1
After	1777	37.4	2408	40.1	2211	40.4	2550	36.6

Values are numbers and percentages unless otherwise stated. To preserve patient anonymity following Danish data regulations, cells with < 5 observations are presented as such

*ACE* angiotensin converting enzyme, *ADP* adenosine diphosphate, *ARB* angiotensin-II receptor blocker, *BMI* body mass index, *CAD* coronary artery disease, *eGFR* estimated glomerular filtration rate, *IQR* inter-quartile range, *VD* vessel disease

Statin treatment after CAG was around 90% for all groups. The primary choice of statin changed from simvastatin to the more potent atorvastatin (Additional file 1: Tables S1 and S2) during the study period. For example, simvastatin and atorvastatin were used in 74% and 12% of patients with diabetes in 2004–2006, but these percentages had changed to 34% and 47% in 2013–2016. The use of antihypertensive drugs also changed over time but in a more heterogenous way. The use of beta-blockers, ACE inhibitors, and thiazides decreased, the use of ARBs increased, and the use of calcium channel blockers remained stable. Finally, in the diabetes group, insulin treatment decreased from the first to the last study interval while use of non-insulin anti-diabetic medication increased.

### Clinical outcomes

**Fig. 2:**
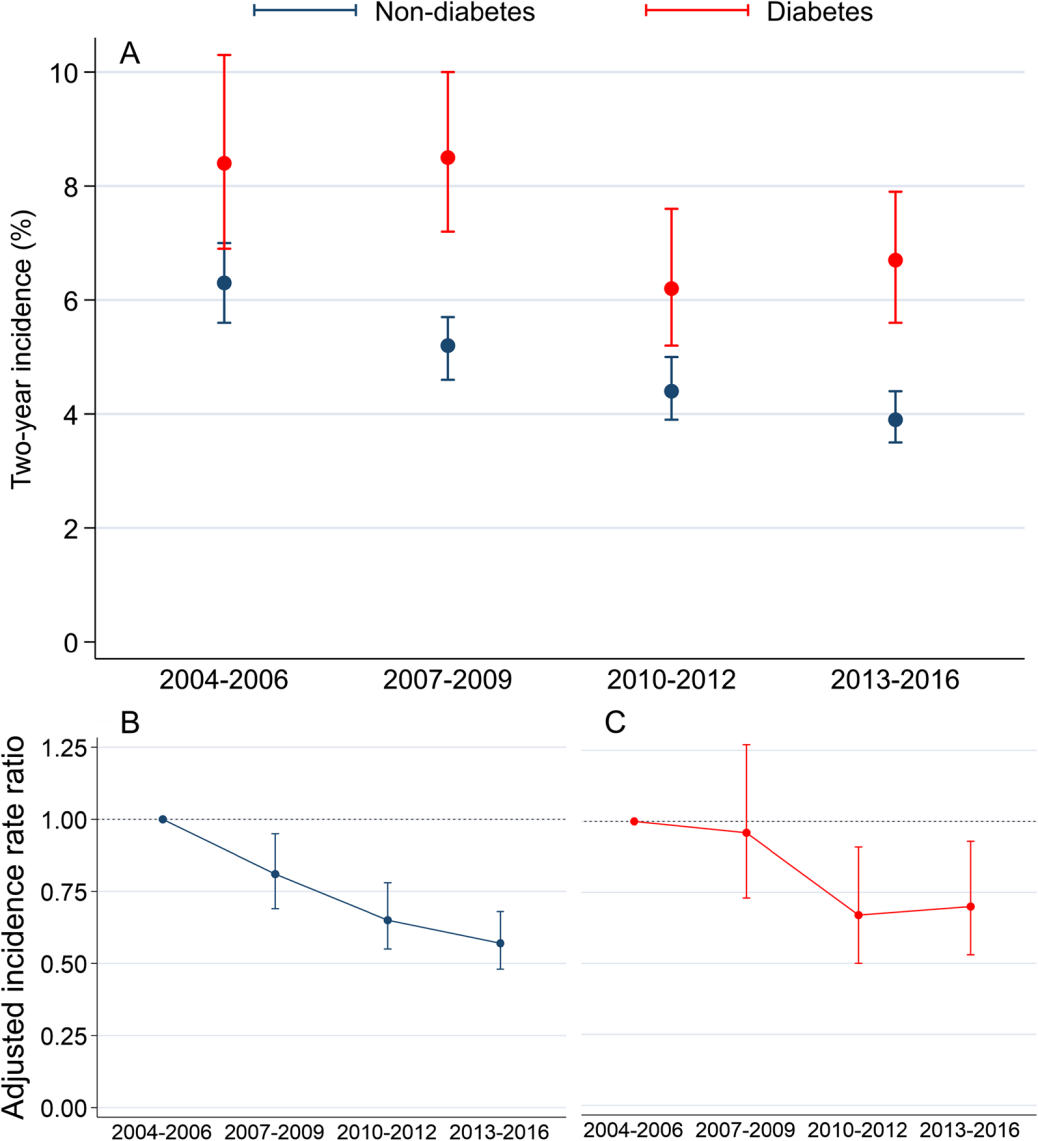
Two-year risks of MACE with 95% confidence intervals (**a**) and adjusted IRR in non-diabetes (**b**) and in diabetes (**c**) patients from 2004 to 2016

Tables 3 (diabetes) and 4 (non-diabetes) report the two-year absolute and relative risks for the four study intervals and is graphically illustrated in Fig. 2. The risk of MACE decreased among patients with diabetes (8.4–6.7%, adjusted incidence rate ratios (aIRR) 0.70, 95% CI 0.53–0.93) and patients without diabetes (6.3–3.9%, aIRR 0.57, 95% CI 0.48–0.68). The two-year risk of MACE remained around 2.5% higher among patients with diabetes in comparison to patients without diabetes through all study intervals (Additional file 1: Table S3). The results were consistent in both patients below and above 70 years (Additional file 1: Tables S8 and S9). Men had 1–2% higher absolute risk of MACE compared to women in patients without diabetes, whereas sex differences was less pronounced among patients with diabetes (Additional file 1: Tables S6 and S7). Both men and women had reductions in MACE through the study period in accordance with our main analysis. In the diabetes group, the MACE reduction was primarily caused by halving the risk of ischemic stroke while relatively smaller, and statistically insignificant, reductions of MI and all-cause death were found. Similar results were found for patients without diabetes. These results were robust when we restricted the analyses to patients with obstructive CAD (i.e., excluding those with diffuse CAD) (Additional file 1: Tables S4 and S5).

**Table 3:** Two-year risk of adverse cardiovascular outcomes after coronary angiography in elective diabetes patients with chronic coronary syndrome

	Patients	Events	Two-year cumultive incidence proportion (95% CI)	Unadjusted IRR (95% CI)	Adjusted IRR* (95% CI)
*MACE*					
2004–2006	1066	89	8.4% (6.9–10.3)	Reference	Reference
2007–2009	1507	126	8.5% (7.2–10.0)	1.01 (0.77–1.33)	0.96 (0.73–1.27)
2010–2012	1523	94	6.2% (5.2–7.6)	0.73 (0.55–1.98)	0.67 (0.50–0.91)
2013–2016	1835	121	6.7% (5.6–7.9)	0.78 (0.59–1.03)	0.70 (0.53–0.93)
*Myocardial infarction*					
2004–2006	1066	42	4.0% (3.0–5.4)	Reference	Reference
2007–2009	1507	57	3.9% (3.0–5.0)	0.97 (0.65–1.45)	0.96 (0.64–1.43)
2010–2012	1523	61	4.1% (3.2–5.2)	1.02 (0.69–1.51)	0.97 (0.65–1.44)
2013–2016	1835	66	3.7% (2.9–4.7)	0.91 (0.61–1.34)	0.85 (0.57–1.25)
*Ischemic stroke*					
2004–2006	1066	36	3.4% (2.5–4.7)	Reference	Reference
2007–2009	1507	40	2.7% (2.0–3.7)	0.79 (0.50–1.24)	0.76 (0.48–1.21)
2010–2012	1523	24	1.6% (1.1–2.4)	0.46 (0.27–0.78)	0.40 (0.24–0.69)
2013–2016	1835	35	2.0% (1.4–2.7)	0.56 (0.35–0.89)	0.47 (0.29–0.76)
*Cardiac death*					
2004–2006	1066	24	2.3% (1.5–3.4)	Reference	Reference
2007–2009	1507	41	2.8% (2.1–3.8)	1.22 (0.74–2.20)	1.12 (0.67–1.87)
2010–2012	1523	17	1.1% (0.7–1.8)	0.50 (0.27–0.92)	0.45 (0.24–0.85)
2013–2016	1835	30	1.7% (1.2–2.4)	0.73 (0.42–1.24)	0.65 (0.38–1.14)
*Death*					
2004–2006	1066	64	6.0% (4.7–7.6)	Reference	Reference
2007–2009	1507	115	7.6% (6.4–9.1)	1.28 (0.95–1.74)	1.21 (0.89–1.64)
2010–2012	1523	85	5.6% (4.5–6.9)	0.93 (0.67–1.28)	0.84 (0.61–1.17)
2013–2016	1835	97	5.3% (4.4–6.4)	0.88 (0.64–1.21)	0.78 (0.56–1.06)

*Adjusted for sex, age, smoking, hypertension, previous ischemic stroke, peripheral artery disease, statin treatment, antiplatelet treatment, and oral anti-coagulant treatment. Ischemic stroke and death were additionally adjusted for atrial fibrillation and heart failure

**Table 4:** Two-year risk of adverse cardiovascular outcomes after coronary angiography in elective non-diabetes patients with chronic coronary syndrome

	Patients	Events	Two-year cumulative incidence proportion (95% CI)	Unadjusted IRR (95% CI)	Adjusted IRR* (95% CI)
*MACE*					
2004–2006	4847	302	6.3% (5.6–7.0)	Reference	Reference
2007–2009	6104	312	5.2% (4.6–5.7)	0.81 (0.69–0.96)	0.81 (0.69–0.95)
2010–2012	5547	243	4.2% (3.9–5.0)	0.69 (0.59–0.82)	0.65 (0.55–0.78)
2013–2016	7042	272	3.9% (3.5–4.4)	0.61 (0.52–0.72)	0.57 (0.48–0.68)
*Myocardial infarction*					
2004–2006	4847	154	3.2% (2.8–3.8)	Reference	Reference
2007–2009	6104	173	2.9% (2.5–3.3)	0.89 (0.71–1.10)	0.89 (0.72–1.11)
2010–2012	5547	125	2.3% (1.0–2.7)	0.70 (0.55–0.89)	0.68 (0.54–0.87)
2013–2016	7042	175	2.5% (2.2–2.9)	0.77 (0.62–0.96)	0.75 (0.60–0.93)
*Ischemic stroke*					
2004–2006	4847	72	1.5% (1.2–1.9)	Reference	Reference
2007–2009	6104	69	1.2% (0.9–1.5)	0.76 (0.54–1.05)	0.71 (0.51–0.99)
2010–2012	5547	64	1.2% (0.9–1.5)	0.77 (0.55–1.07)	0.65 (0.46–0.92)
2013–2016	7042	62	0.9% (0.7–1.1)	0.58 (0.42–0.82)	0.48 (0.34–0.68)
*Cardiac death*					
2004–2006	4847	102	2.1% (1.8–2.6)	Reference	Reference
2007–2009	6104	99	1.6% (1.4–2.0)	0.77 (0.58–1.01)	0.77 (0.58–1.87)
2010–2012	5547	65	1.2% (0.9–1.5)	0.55 (0.40–0.76)	0.52 (0.38–0.71)
2013–2016	7042	50	0.7% (0.5–0.9)	0.33 (0.24–0.47)	0.31 (0.22–0.44)
*Death*					
2004–2006	4847	229	4.7% (4.2–5.4)	Reference	Reference
2007–2009	6104	270	4.4% (3.9–5.0)	0.93 (0.78–1.11)	0.95 (0.79–1.13)
2010–2012	5547	219	4.0% (3.5–4.5)	0.83 (0.69–1.00)	0.80 (0.66–0.97)
2013–2016	7042	216	3.1% (2.7–3.5)	0.64 (0.53–0.77)	0.61 (0.51–0.74)

*Adjusted for sex, age, smoking, hypertension, previous ischemic stroke, peripheral artery disease, statin treatment, antiplatelet treatment, and oral anti-coagulant treatment. Ischemic stroke and death were additionally adjusted for atrial fibrillation and heart failure

### Revascularization

The revascularization rates decreased within the first three months after index CAG for both PCI and CABG from the first to the last study interval (Table 5). This was found for both diabetes and non-diabetes patients. Similar results were found when analysing revascularization for the entire two-year study period (Additional file 1: Table S10). However, when restricting the analysis to patients with obstructive CAD, we found a change in the revascularization pattern with more patients being treated with PCI and fewer patients with CABG over time, a finding that was consistent among diabetes and non-diabetes patients (Additional file 1: Table S10).

**Table 5:** Coronary revascularization among diabetes and non-diabetes patients within three months after index coronary angiography

	Patients	Events	3-month cumulative incidence proportions (95% CI)	Unadjusted IRR (95% CI)	Adjusted IRR* (95% CI)
**Diabetes**					
*Percutaneous coronary intervention*					
2004–2006	1066	494	46.3% (43.4–49.3)	Reference	Reference
2007–2009	1507	598	39.7% (37.2–42.2)	0.76 (0.65–0.89)	0.76 (0.65–0.90)
2010–2012	1523	603	39.6% (37.1–42.1)	0.76 (0.65–0.89)	0.76 (0.65–0.89)
2013–2016	1835	696	37.9% (35.7–40.2)	0.71 (0.61–0.82)	0.70 (0.60–0.82)
*Coronary artery bypass grafting*					
2004–2006	1066	240	22.5% (20.0–25.0)	Reference	Reference
2007–2009	1507	273	18.1% (16.2–20.1)	0.76 (0.63–0.92)	0.76 (0.62–0.92)
2010–2012	1523	245	16.1% (14.2–17.9)	0.66 (0.54–0.80)	0.65 (0.53–0.80)
2013–2016	1835	342	18.6% (16.9–20.4)	0.79 (0.65–0.95)	0.80 (0.66–0.96)
**Non-diabetes**					
*Percutaneous coronary intervention*					
2004–2006	4847	2403	49.6% (48.2–51.0)	Reference	Reference
2007–2009	6104	2766	45.3% (44.1–46.6)	0.84 (0.78–0.91)	0.85 (0.79–0.92)
2010–2012	5547	2396	43.2% (41.9–44.5)	0.77 (0.72–0.84)	0.79 (0.73–0.85)
2013–2016	7042	2887	41.0% (39.9–42.2)	0.71 (0.66–0.76)	0.72 (0.67–0.78)
*Coronary artery bypass grafting*					
2004–2006	4847	1026	21.2% (20.0–22.3)	Reference	Reference
2007–2009	6104	1013	16.6% (15.7–17.5)	0.74 (0.67–0.82)	0.74 (0.67–0.82)
2010–2012	5547	950	17.1% (16.1–18.1)	0.77 (0.70–0.85)	0.78 (0.71–0.86)
2013–2016	7042	1115	15.8% (15.0–16.7)	0.70 (0.64–0.77)	0.71 (0.64–0.78)

*Adjusted for sex, age, smoking, hypertension, previous ischemic stroke, peripheral artery disease, statin treatment, antiplatelet treatment, oral anti-coagulant treatment

## Discussion

### Statement of principal findings

Our main finding is that the two-year relative MACE risk decreased by 30% in patients with diabetes who presented with chronic coronary syndrome in Denmark from 2004 to 2016. This result was primarily caused by a reduction in ischemic stroke. However, since even larger relative and absolute risk reductions were observed among patients without diabetes, the gap between patients with and without diabetes did not change.

### Diabetes

In the diabetes group, the absolute two-year risk of MACE decreased by 1.7% from 2004–2006 to 2013–2016. This is likely the result of several guideline-directed initiatives implemented in Denmark within the inclusion period. First, the focus on cardiovascular prevention has increased in diabetes patients where an intensified multifactorial intervention with tight regulation of blood glucose, blood pressure, and lipid-levels has proven to lower cardiovascular risk in diabetes patients [1]. Although approximately 90% of diabetes patients received statin treatment after the CAG, we observed a change in the primary choice of statin from simvastatin to the more potent atorvastatin during the study period, i.e*.*, suggesting intensified lipid-lowering treatment [22]. Second, CABG is superior to PCI in patients with diabetes plus obstructive multivessel disease [10, 23]. In the diabetes cohort, the use of PCI was reduced by an absolute 8% while CABG decreased from 23% in 2004–2006 and remained stable around 18% throughout the last three study intervals. This suggests adherence to clinical guidelines in a time where FFR often led to downgrading of multivessel disease and where PCI in general tended to be preferred over CABG. Third, newer-generation DES have replaced bare-metal stents and first-generation DES during the study period. Newer-generation DES reduce MACE rates up to five years after PCI compared with first-generation DES [9, 24] and the two-year follow-up period may be too short to capture the benefit of newer-generation DES. Newer-generation DES also displayed higher safety in patients with diabetes [25, 26]. However, the main reduction among diabetes patients was caused by reduced risk of ischemic stroke while patients without diabetes had reduced risk of all cardiovascular events.

### Obstructive CAD

Fewer patients were classified as having obstructive multivessel CAD while more were classified as diffuse non-obstructive CAD. Theoretically, this can be explained by earlier detection of CAD (lead time bias), delayed progression of CAD, or changed perception of CAD significance. Since the median age increased from 66 years in 2004–2006 to 68 years in 2013–2016, we find it unlikely that lead time bias and delayed progression of CAD are the main explanations for the observed reduced rates of multi-vessel disease. In contrast, the gradual implementation of intracoronary physiology measurements, such as FFR, to assist visual assessment of intermediate stenoses has undoubtedly led to downgrading of CAD severity since visual assessment alone tend to overestimate disease significance [27]. Importantly, MACE also decreased when we restricted our analyses to only include patients with obstructive CAD, i.e., the reduction of events was not explained by inclusion of more patients with diffuse CAD due to a changed registration pattern of non-obstructive CAD. Furthermore, the reduced cardiovascular risk among patients with obstructive CAD is presumably an underestimation of the actual reduced risk as we expect that some of the patients with “obstructive” CAD in the earlier study periods would have been classified as non-obstructive in the later periods when FFR became a standard tool in our daily clinical practice. Finally, in our sensitivity analysis of patients with *obstructive* CAD, it is noteworthy that the “downgrading” of CAD severity led to more use of PCI and less use of CABG among both diabetes and non-diabetes patients.

### Comparison with other work

We have not been able to identify previous studies looking at changes in cardiovascular outcomes among patients with diabetes and chronic coronary syndrome. Our results, however, are in accordance with our previous study looking at improvements in 7-years outcomes among Danish patients with new-onset diabetes from 1996 to 2011 [28] as well as a Swedish study examining outcomes among patients with prevalent diabetes from 1998 to 2014 [29]. Moreover, two Swedish studies compared outcomes for all patients with acute coronary syndrome from 1995 to 2014 [30, 31] but differed concerning inclusion criteria (chronic vs acute coronary syndrome), study period, and lack of stratification based on presence of diabetes. Still, the studies share similarities by including a Scandinavian cohort treated in a national, tax-payer funded, public health care system, and the overall trends with reduced cardiovascular risk over the study period.

### Clinical implications

It was recently shown that the risk of adverse cardiovascular events among patients with new-onset diabetes without previous cardiovascular disease decreased markedly from 1996 to 2011, drawing close to the cardiovascular risk of patients without diabetes [28]. In our study, we found that the relative risk of MACE decreased by 30% in patients with diabetes from 2004–2016, although their risk remained substantially increased compared to patients without diabetes. Therefore, an early and aggressive treatment strategy (i.e. cholesterol lowering drugs, blood pressure management, exercise, diet counseling, and smoking cessation) before the development of cardiovascular disease seems essential in order to minimize cardiovascular risk among diabetes patients, and such a multifactorial strategy, as documented by fewer active smokers and more use of high-intensity statins, likely played a role for the 30% risk reduction observed among the diabetes patients.

### Limitations

Our study has some limitations to consider. The definition of MI was revised in 2007 and again in 2012 following the introduction of new high-sensitive cardiac troponin assays [32, 33]. Lowering of the 99^th^ percentile upper normal reference limit due to improved biomarker sensitivity enabled smaller increases in troponin levels to meet the MI criteria. The lower MI diagnosis threshold in the later examination year intervals may underestimate the true reduction in MI during the study period [34].

Due to lack of biochemical data on our study group, we were unable to differentiate between prediabetic patients and normoglycemic patients in the non-diabetes group and investigate potential differences in cardiovascular outcomes [35, 36].

It is difficult to distinguish between type 1 and 2 diabetes based on registries alone. However, type 2 diabetes is by far the most common diabetes type in this age group and our results are thus mainly representative of patients with type 2 diabetes. As such, our results may not be representative for type 1 diabetes patients.

All studies assessing changes over time are limited by the fact that multiple changes have taken place during a long study period. While the main finding is that a large relative risk reduction was observed, which thereby shows that cardiovascular risk reduction is possible even in a 12-year period, it is difficult to define a specific cause.

Finally, our results were obtained in a tax-payer funded, public health care system with equal access for all citizens, and the external validity to societies with greater socioeconomic disparities needs confirmation.

### Conclusion

In Denmark from 2004 to 2016, we found a reduced two-year risk of MACE among both diabetes and non-diabetes patients with chronic coronary syndrome. However, despite improvements in cardiovascular risk and changed treatment patterns, diabetes patients with chronic coronary syndrome remain at higher risk of MACE than patients without diabetes. An intensive, multifactorial treatment strategy before the development of cardiovascular disease is essential in order to minimize cardiovascular risk among diabetes patients.

## Supplementary Information

**Additional file 1: Table S1 and Table S2** show changes in statin and ADP inhibitor treatment in diabetes and non-diabetes patients from 2004 to 2016. Table S3 compares two-year risks of major adverse cardiovascular events between diabetes and non-diabetes patients from 2004 to 2016. Table S4 and S5 show two-year risks of major adverse cardiovascular events in diabetes and non-diabetes patients with chronic coronary disease and obstructive coronary disease. Table S6 and S7 show two-year risks of major adverse cardiovascular events in diabetes and non-diabetes patients stratified by sex. Table S8 and S9 show two-year risks of major adverse cardiovascular events in diabetes and non-diabetes patients stratified by age above or below 70 years. Table S10 shows two-year risks of coronary revascularization after coronary angiography in diabetes and non-diabetes patients with any coronary artery disease and in diabetes and non-diabetes patients with obstructive coronary artery disease.

## Data Availability

According to Danish data protection regulations, data cannot be made publicly available.

## Abbreviations

MI: Myocardial infarction
CAD: Coronary artery disease
PCI: Percutaneous coronary intervention
FFR: Fractional flow reserve
CAG: Coronary angiography
CABG: Coronary artery bypass grafting
BMI: Body mass index
ADP: Adenosine diphosphate
ACE: Angiotensin-converting enzyme
ARB: Angiotensin II receptor blocker
MACE: Major adverse cardiovascular event
ATC: Anatomic Therapeutic Chemical
ICD-10: International Classification of Diseases 10
IRR: Incidence rate ratio
aIRR: Adjusted incidence rate ratio
DES: Drug-eluting stent
