# Supplementary material for: Cardiovascular risk in patients with and without diabetes presenting with chronic coronary syndrome in 2004–2016
Source: BMC Cardiovasc Disord. 2021 Dec 4;21:579. doi: 10.1186/s12872-021-02312-y (PMC8642966; doi:10.1186/s12872-021-02312-y)
Supplement: Supplementary file 1 — Additional file 1: Table S1 and Table S2 show changes in statin and ADP inhibitor treatment in diabetes and non-diabetes patients from 2004 to 2016. Table S3 compares two-year risks of major adverse cardiovascular events between diabetes and non-diabetes patients from 2004 to 2016. Table S4 and S5 show two-year risks of major adverse cardiovascular events in diabetes and non-diabetes patients with chronic coronary disease and obstructive coronary disease. Table S6 and S7 show two-year risks of major adverse cardiovascular events in diabetes and non-diabetes patients stratified by sex. Table S8 and S9 show two-year risks of major adverse cardiovascular events in diabetes and non-diabetes patients stratified by age above or below 70 years. Table S10 shows two-year risks of coronary revascularization after coronary angiography in diabetes and non-diabetes patients with any coronary artery disease and in diabetes and non-diabetes patients with obstructive coronary artery disease. [file 12872_2021_2312_MOESM1_ESM.docx]

Supplemental Material

| **Table S1.** Changes in six-month post-procedural statin and adenosine diphosphate (ADP) inhibitor treatment in diabetes patients with more than six months of follow-up (n=5,832). | | | | | | | | | |
| --- | --- | --- | --- | --- | --- | --- | --- | --- | --- |
|  | | 2004-2006  n=1,049 | | 2007-2009  n=1,476 | | 2010-2012  n=1,503 | | 2013-2016  n=1,804 | |
| *Statin* | |  |  |  |  |  |  |  |  |
|  | None | 75 | 7.1 | 125 | 8.5 | 139 | 9.2 | 201 | 11.1 |
|  | Simvastatin | 778 | 74.2 | 1,063 | 72.0 | 1,004 | 66.8 | 610 | 33.8 |
|  | Atorvastatin | 129 | 12.3 | 195 | 13.2 | 217 | 14.4 | 844 | 46.8 |
|  | Rosuvastatin | 229 | 2.8 | 68 | 4.6 | 123 | 8.2 | 130 | 7.2 |
|  | Other* | 38 | 3.6 | 25 | 1.7 | 20 | 1.3 | 19 | 1.1 |
| *ADP-inhibitor* | |  |  |  |  |  |  |  |  |
|  | None | 529 | 50.4 | 821 | 55.6 | 850 | 56.6 | 1,037 | 57.5 |
|  | Clopidogrel | 520 | 49.6 | 655 | 44.4 | 631 | 42.0 | 737 | 40.9 |
|  | Other† | 0 | 0.0 | 0 | 0.0 | 22 | 1.5 | 30 | 1.7 |
| *Lovastatin, pravastatin, or fluvastatin.  †Prasugrel or ticagrelor. | | | | | | | | | |

| **Table S2.** Changes in six-month post-procedural statin and adenosine diphosphate (ADP) inhibitor treatment in non-diabetes patients with more than six months of follow-up (n=23,191). | | | | | | | | | | | | | |  |
| --- | --- | --- | --- | --- | --- | --- | --- | --- | --- | --- | --- | --- | --- | --- |
|  | | 2004-2006  n=4,755 | | 2007-2009  n=5,999 | | | 2010-2012  n=5,475 | | | | 2013-2016  n=6,962 | | | |
| *Statin* | |  |  | |  |  | |  |  |  | |  |  |  |
|  | None | 397 | 8.3 | | 520 | 8.7 | | 566 | 10.3 | 716 | | 10.3 |  |  |
|  | Simvastatin | 3,744 | 78.7 | | 4,620 | 77.0 | | 3,836 | 70.1 | 1,601 | | 23.0 |  |  |
|  | Atorvastatin | 364 | 7.7 | | 518 | 8.6 | | 690 | 12.6 | 4,248 | | 61.0 |  |  |
|  | Rosuvastatin | 112 | 2.4 | | 237 | 4.0 | | 318 | 5.8 | 351 | | 5.0 |  |  |
|  | Other* | 138 | 2.9 | | 104 | 1.7 | | 65 | 1.2 | 46 | | 0.7 |  |  |
| *ADP-inhibitor* | |  |  | |  |  | |  |  |  | |  |  |  |
|  | None | 2,215 | 46.6 | | 3,036 | 50.6 | | 2,936 | 53.6 | 3,708 | | 53.3 |  |  |
|  | Clopidogrel | 2,450 | 53.4 | | 2,962 | 49.4 | | 2,460 | 44.9 | 3,123 | | 44.9 |  |  |
|  | Other† | 0 | 0.0 | | <5 | <0.01 | | 79 | 1.4 | 131 | | 1.9 |  |  |
| To preserve patient anonymity following Danish data protection regulations, cells <5 observation are presented as such.  *Lovastatin, pravastatin, and fluvastatin.  †Prasugrel or ticagrelor. | | | | | | | | | | | | |  |  |

| **Table S3.** Two-year risk of major adverse cardiovascular events in diabetes patients with chronic coronary syndrome compared with non-diabetes patients with chronic coronary syndrome. | | | | | | |
| --- | --- | --- | --- | --- | --- | --- |
|  | Diabetes (events) | Non-diabetes (events) | Two-year risk difference  (95% CI) | Unadjusted IRR (95 % CI) | Adjusted IRR* (95 % CI) |  |
| 2004-2006 | 1,066 (89) | 4,847 (302) | 2.1% (0.3-3.9) | 1.36 (1.07-1.73) | 1.27 (0.99-1.63) |  |
| 2007-2009 | 1,507 (126) | 6,104 (312) | 3.3% (1.8-4.8) | 1.69 (1.37-2.08) | 1.60 (1.29-2.00) |  |
| 2010-2012 | 1,523 (94) | 5,547 (243) | 1.8% (0.5-3.1) | 1.44 (1.13-1.83) | 1.34 (1.04-1.73) |  |
| 2013-2016 | 1,835 (272) | 7,042 (272) | 2.8% (1.5-4.0) | 1.74 (1.40-2.15) | 1.56 (1.24-1.95) |  |
| *Adjusted for sex, age, smoking, hypertension, previous ischemic stroke, peripheral artery disease, statin treatment, antiplatelet treatment, and oral anti-coagulant treatment. | | | | | | |

| **Table S4.** Two-year risk of major adverse cardiovascular events (MACE) after coronary angiography in elective diabetes patients with chronic coronary syndrome and obstructive coronary artery disease (n=4,781). | | | |
| --- | --- | --- | --- |
|  | | Unadjusted IRR (95 % CI) | Adjusted IRR* (95 % CI) |
| *MACE* | | | |
|  | 2004-2006 | reference | reference |
|  | 2007-2009 | 1.10 (0.83-1.46) | 1.05 (0.79-1.39) |
|  | 2010-2012 | 0.87 (0.65-1.18) | 0.77 (0.56-1.05) |
|  | 2013-2016 | 0.92 (0.69-1.22) | 0.81 (0.60-1.08) |
| *Myocardial infarction* | | |  |
|  | 2004-2006 | reference | reference |
|  | 2007-2009 | 1.12 (0.74-1.69) | 1.11 (0.73-1.67) |
|  | 2010-2012 | 1.21 (0.80-1.83) | 1.13 (0.75-1.72) |
|  | 2013-2016 | 1.12 (0.75-1.67) | 1.03 (0.69-1.54) |
| *Ischemic stroke* | |  |  |
|  | 2004-2006 | reference | reference |
|  | 2007-2009 | 0.85 (0.53-1.35) | 0.80 (0.50-1.30) |
|  | 2010-2012 | 0.57 (0.34-0.97) | 0.47 (0.27-0.81) |
|  | 2013-2016 | 0.59 (0.36-0.97) | 0.49 (0.29-0.81) |
| *Cardiac death* | |  |  |
|  | 2004-2006 | reference | reference |
|  | 2007-2009 | 1.28 (0.76-2.13) | 1.16 (0.69-1.96) |
|  | 2010-2012 | 0.62 (0.22-1.15) | 0.54 (0.28-1.02) |
|  | 2013-2016 | 0.90 (0.52-0.59) | 0.79 (0.45-1.37) |
| *Death* | | | |
|  | 2004-2006 | reference | Reference |
|  | 2007-2009 | 1.40 (0.98-1.83) | 1.24 (0.91-1.70) |
|  | 2010-2012 | 0.98 (0.70-1.38) | 0.85 (0.60-1.20) |
|  | 2013-2016 | 0.96 (0.69-1.33) | 0.83 (0.59-1.15) |
| *Adjusted for sex, age, smoking, hypertension, previous ischemic stroke, peripheral artery disease, statin treatment, antiplatelet treatment, and oral anti-coagulant treatment. Ischemic stroke and death were additionally adjusted for atrial fibrillation and heart failure. | | | |

| **Table S5.** Two-year risk of major adverse cardiovascular events (MACE) after coronary angiography in elective non-diabetes patients with chronic coronary disease and obstructive coronary artery disease (n=19,077). | | | |
| --- | --- | --- | --- |
|  | | Unadjusted IRR (95 % CI) | Adjusted IRR* (95 % CI) |
| *MACE* | | | |
|  | 2004-2006 | reference | reference |
|  | 2007-2009 | 0.86 (0.73-1.02) | 0.84 (0.71-0.99) |
|  | 2010-2012 | 0.78 (0.65-0.93) | 0.72 (0.61-0.87) |
|  | 2013-2016 | 0.73 (0.62-0.87) | 0.67 (0.56-0.79) |
| *Myocardial infarction, 0-24 months* | | |  |
|  | 2004-2006 | reference | reference |
|  | 2007-2009 | 0.97 (0.78-1.22) | 0.97 (0.78-1.22) |
|  | 2010-2012 | 0.82 (0.64-1.04) | 0.78 (0.61-1.00) |
|  | 2013-2016 | 0.94 (0.75-1.17) | 0.89 (0.71-1.11) |
| *Ischemic stroke* | |  |  |
|  | 2004-2006 | reference | reference |
|  | 2007-2009 | 0.73 (0.51-1.03) | 0.67 (0.47-0.95) |
|  | 2010-2012 | 0.78 (0.54-1.11) | 0.66 (0.54-0.95) |
|  | 2013-2016 | 0.67 (0.47-0.95) | 0.52 (0.47-0.75) |
| *Cardiac death* | |  |  |
|  | 2004-2006 | reference | reference |
|  | 2007-2009 | 0.83 (0.62-1.10) | 0.81 (0.61-1.08) |
|  | 2010-2012 | 0.65 (0.47-0.90) | 0.60 (0.43-0.82) |
|  | 2013-2016 | 0.42 (0.29-0.59) | 0.38 (0.26-0.54) |
| *Death* | | | |
|  | 2004-2006 | reference | reference |
|  | 2007-2009 | 0.96 (0.80-1.15) | 0.96 (0.80-1.15) |
|  | 2010-2012 | 0.91 (0.75-1.11) | 0.86 (0.70-1.04) |
|  | 2013-2016 | 0.65 (0.54-0.80) | 0.60 (0.49-0.74) |
| *Adjusted for sex, age, smoking, hypertension, previous ischemic stroke, peripheral artery disease, statin treatment, antiplatelet treatment, and oral anti-coagulant treatment. Ischemic stroke and death were additionally adjusted for atrial fibrillation and heart failure. | | | |

| **Table S6.** Two-year risk of major adverse cardiovascular events after coronary angiography in elective diabetes patients with stable angina pectoris by sex. | | | | | | |  |
| --- | --- | --- | --- | --- | --- | --- | --- |
|  | | Patients | Events | Two-year incidence  (95% CI) | Unadjusted IRR (95 % CI) | Adjusted IRR* (95 % CI) | |
| *Men* | |  |  |  |  |  | |
|  | 2004-2006 | 780 | 61 | 7.9% (6.2-10.0) | reference | reference | |
|  | 2007-2009 | 1,061 | 94 | 9.0% (7.4-10.9) | 1.15 (0.83-1.60) | 1.08 (0.77-1.50) | |
|  | 2010-2012 | 1,058 | 70 | 6.7% (5.3-8.4) | 0.84 (0.60-1.19) | 0.75 (0.53-1.07) | |
|  | 2013-2016 | 1,301 | 85 | 6.6% (5.4-8.1) | 0.83 (0.59-1.15) | 0.74 (0.53-1.03) | |
| *Women* | | | |  |  |  | |
|  | 2004-2006 | 286 | 28 | 9.9% (6.9-13.9) | reference | reference | |
|  | 2007-2009 | 446 | 32 | 7.2% (5.2-10.1) | 0.72 (0.43-1.21) | 0.72 (0.42-1.23) | |
|  | 2010-2012 | 465 | 24 | 5.2% (3.5-7.7) | 0.52 (0.30-0.90) | 0.51 (0.29-0.91) | |
|  | 2013-2016 | 534 | 36 | 6.8% (5.0-9.3) | 0.67 (0.40-1.11) | 0.63 (0.38-1.06) | |
| *Adjusted for sex, smoking, hypertension, previous ischemic stroke, peripheral artery disease, statin treatment, antiplatelet treatment, and oral anti-coagulant treatment. In ischemic stroke and death, additionally adjusted for atrial fibrillation and heart failure. | | | | | | |  |

| **Table S7.** Two-year risk of major adverse cardiovascular events after coronary angiography in elective non-diabetes patients with stable angina pectoris by sex. | | | | | | | | | | | |
| --- | --- | --- | --- | --- | --- | --- | --- | --- | --- | --- | --- |
|  | | Patients | | | Events | | Two-year incidence  (95% CI) | | Unadjusted IRR (95 % CI) | | Adjusted IRR* (95 % CI) |
| *Men* | |  | | |  | |  | |  | |  |
|  | 2004-2006 | 3,554 | | | 240 | | 6.8% (6.0-7.7) | | reference | | reference |
|  | 2007-2009 | 4,279 | | | 228 | | 5.4% (4.7-6.1) | | 0.78 (0.65-0.94) | | 0.77 (0.64-0.93) |
|  | 2010-2012 | 3,732 | | | 172 | | 4.7% (4.0-5.4) | | 0.67 (0.55-0.82) | | 0.62 (0.51-0.76) |
|  | 2013-2016 | 4,843 | | | 203 | | 4.2% (3.7-4.8) | | 0.61 (0.51-0.74) | | 0.55 (0.46-0.67) |
| *Women* | | | | | |  | |  | |  | |
|  | 2004-2006 | | 1,293 | 62 | | 4.8% (3.8-6.2) | | reference | | reference | |
|  | 2007-2009 | | 1,825 | 84 | | 4.6% (3.8-5.7) | | 0.96 (0.69-1.34) | | 0.92 (0.66-1.28) | |
|  | 2010-2012 | | 1,815 | 71 | | 3.9% (3.1-4.9) | | 0.81 (0.58-1.15) | | 0.75 (0.53-1.07) | |
|  | 2013-2016 | | 2,199 | 69 | | 3.2% (2.5-4.0) | | 0.65 (0.46-0.92) | | 0.61 (0.43-0.87) | |
| *Adjusted for sex, smoking, hypertension, previous ischemic stroke, peripheral artery disease, statin treatment, antiplatelet treatment, and oral anti-coagulant treatment. In ischemic stroke and death, additionally adjusted for atrial fibrillation and heart failure. | | | | | | | | | | | |

| **Table S8.** Two-year risk of major adverse cardiovascular events after coronary angiography in elective diabetes patients with stable angina pectoris by age group | | | | | | | | | | |  |
| --- | --- | --- | --- | --- | --- | --- | --- | --- | --- | --- | --- |
|  | | Patients | | Events | | 2-year incidence  (95% CI) | Unadjusted IRR (95 % CI) | | Adjusted IRR* (95 % CI) | | |
| *<70 years* | |  | |  | |  |  | |  | | |
|  | 2004-2006 | 714 | | 53 | | 7.5% (5.8-9.7) | reference | | reference | | |
|  | 2007-2009 | 987 | | 57 | | 5.8% (4.5-7.5) | 0.77 (0.53-1.12) | | 0.76 (0.51-1.11) | | |
|  | 2010-2012 | 962 | | 52 | | 5.4% (4.2-7.1) | 0.72 (0.49-1.06) | | 0.67 (0.45-1.00) | | |
|  | 2013-2016 | 1,049 | | 54 | | 5.2% (4.0-6.7) | 0.68 (0.46-1.00) | | 0.66 (0.44-0.97) | | |
| *≥70 years* | | | | |  | |  | |  | | |
|  | 2004-2006 | 352 | 36 | | 10.3% (7.6-14.1) | | | reference | reference |  |  |
|  | 2007-2009 | 520 | 69 | | 13.5% (10.9-16.8) | | | 1.35 (0.90-2.03) | 1.27 (0.84-1.93) |  |  |
|  | 2010-2012 | 561 | 42 | | 7.6% (5.7-10.2) | | | 0.73 (0.46-1.15) | 0.69 (0.43-1.10) |  |  |
|  | 2013-2016 | 786 | 67 | | 8.7% (6.9-10.9) | | | 0.82 (0.54-1.24) | 0.79 (0.52-1.20) |  |  |
| *Adjusted for sex, smoking, hypertension, previous ischemic stroke, peripheral artery disease, statin treatment, antiplatelet treatment, and oral anti-coagulant treatment. In ischemic stroke and death, additionally adjusted for atrial fibrillation and heart failure. | | | | | | | | | | |  |

| **Table S9.** Two-year risk of major adverse cardiovascular events after coronary angiography in elective non-diabetes patients with stable angina pectoris by age group | | | | | | | | | |  |
| --- | --- | --- | --- | --- | --- | --- | --- | --- | --- | --- |
|  | | Patients | Events | | 2-year incidence  (95% CI) | | Unadjusted IRR (95 % CI) | Adjusted IRR* (95 % CI) | |  |
| *<70 years* | |  |  | |  | |  |  | |  |
|  | 2004-2006 | 3,016 | 142 | | 4.7% (4.0-5.6) | | reference | reference | |  |
|  | 2007-2009 | 3,714 | 128 | | 3.5% (2.9-4.1) | | 0.73 (0.57-0.92) | 0.72 (0.56-0.91) | |  |
|  | 2010-2012 | 3,298 | 103 | | 3.1% (2.6-3.8) | | 0.65 (0.51-0.84) | 0.63 (0.49-0.82) | |  |
|  | 2013-2016 | 4,203 | 116 | | 2.8% (2.3-3.3) | | 0.58 (0.45-0.74) | 0.58 (0.45-0.74) | |  |
| *≥70 years* | | | |  | |  | | |  |  |
|  | 2004-2006 | 1,831 | 160 | | 8.9% (7.7-10.3) | | reference | reference | | |
|  | 2007-2009 | 2,390 | 184 | | 7.8% (6.8-9.0) | | 0.88 (0.71-1.08) | 0.88 (0.71-1.09) | | |
|  | 2010-2012 | 2,249 | 140 | | 6.3% (5.4-7.4) | | 0.70 (0.56-0.88) | 0.69 (0.55-0.87) | | |
|  | 2013-2016 | 2,839 | 156 | | 5.6% (4.8-6.5) | | 0.62 (0.49-0.77) | 0.59 (0.47-0.74) | | |
| *Adjusted for sex, smoking, hypertension, previous ischemic stroke, peripheral artery disease, statin treatment, antiplatelet treatment, and oral anti-coagulant treatment. In ischemic stroke and death, additionally adjusted for atrial fibrillation and heart failure. | | | | | | | | | |  |

| **Table S10.** Two-year risk of coronary revascularization after coronary angiography in diabetes and non-diabetes patients with any coronary artery disease and in diabetes and non-diabetes patients with obstructive coronary artery disease. | | | | | | | | | | |  |
| --- | --- | --- | --- | --- | --- | --- | --- | --- | --- | --- | --- |
|  | | Patients | Events | Two-year incidence  (95% CI) | | Unadjusted IRR (95 % CI) | | Adjusted IRR* (95 % CI) | |  |  |
| **Diabetes** | |  |  |  | |  | |  | |  |  |
| *Percutaneous coronary intervention* | | | |  | |  | |  | |  |  |
|  | 2004-2006 | 1,066 | 525 | 49.5% (46.5-52.5) | | reference | | reference | |  |  |
|  | 2007-2009 | 1,507 | 642 | 42.8% (40.4-45.4) | | 0.78 (0.67-0.91) | | 0.78 (0.67-0.92) | |  |  |
|  | 2010-2012 | 1,523 | 644 | 42.4% (39.9-44.9) | | 0.76 (0.65-0.89) | | 0.76 (0.65-0.89) | |  |  |
|  | 2013-2016 | 1,835 | 748 | 40.9% (38.7-43.2) | | 0.72 (0.62-0.83) | | 0.71 (0.61-0.83) | |  |  |
| *Coronary artery bypass grafting* | | | |  | |  | |  | |  |  |
|  | 2004-2006 | 1,066 | 278 | 26.2% (23.7-29.0) | | reference | | reference | |  |  |
|  | 2007-2009 | 1,507 | 339 | 22.7% (20.6-24.9) | | 0.83 (0.70-1.00) | | 0.83 (0.69-1.00) | |  |  |
|  | 2010-2012 | 1,523 | 262 | 17.3% (15.5-19.3) | | 0.60 (0.50-0.72) | | 0.60 (0.49-0.72) | |  |  |
|  | 2013-2016 | 1,835 | 370 | 20.3% (18.5-22.2) | | 0.73 (0.61-0.86) | | 0.74 (0.62-0.88) | |  |  |
| **Non-diabetes**  *Percutaneous coronary intervention* | | | | | | | | | | |  |
|  | 2004-2006 | 4,847 | 2,527 | | 52.3% (50.9-53.7) | | reference | | reference | | |
|  | 2007-2009 | 6,104 | 2,915 | | 47.9% (46.7-49.2) | | 0.85 (0.79-0.91) | | 0.86 (0.80-0.92) | | |
|  | 2010-2012 | 5,547 | 2,500 | | 45.1% (43.8-46.5) | | 0.76 (0.71-0.82) | | 0.78 (0.73-0.85) | | |
|  | 2013-2016 | 7,042 | 3,027 | | 43.0% (41.9-44.2) | | 0.70 (0.65-0.75) | | 0.71 (0.66-0.77) | | |
| *Coronary artery bypass grafting* | | | | | | | | | | |  |
|  | 2004-2006 | 4,847 | 1,175 | | 24.3% (23.2-25.6) | | reference | | reference | | |
|  | 2007-2009 | 6,104 | 1,203 | | 19.8% (18.8-20.8) | | 0.77 (0.71-0.84) | | 0.77 (0.71-0.85) | | |
|  | 2010-2012 | 5,547 | 1,000 | | 18.1% (17.1-19.1) | | 0.69 (0.63-0.76) | | 0.71 (0.64-0.78) | | |
|  | 2013-2016 | 7,042 | 1,182 | | 16.8% (16.0-17.7) | | 0.64 (0.58-0.69) | | 0.64 (0.59-0.71) | | |
| **Diabetes and obstructive CAD (n=4,781)** | | | | |  | |  | |  | | |
| *Percutaneous coronary intervention* | | | | |  | |  | |  | | |
|  | 2004-2006 | 1,014 | 518 | | 51.3% (48.3-54.4) | | reference | | reference | | |
|  | 2007-2009 | 1,242 | 629 | | 51.0% (48.2-53.8) | | 1.00 (0.85-1.18) | | 1.00 (0.85-1.18) | | |
|  | 2010-2012 | 1,162 | 634 | | 54.7% (51.9-57.6) | | 1.16 (0.98-1.36) | | 1.17 (0.98-1.37) | | |
|  | 2013-2016 | 1,363 | 742 | | 54.6% (52.0-57.3) | | 1.14 (0.97-1.23) | | 1.14 (0.97-1.34) | | |
| *Coronary artery bypass grafting* | | | | |  | |  | |  | | |
|  | 2004-2006 | 1,014 | 277 | | 18.8% (16.7-21.1) | | reference | | reference | | |
|  | 2007-2009 | 1,242 | 335 | | 18.7% (16.8-20.8) | | 1.00 (0.83-1.19) | | 0.88 (0.81-0.95) | | |
|  | 2010-2012 | 1,162 | 262 | | 14.8% (13.1-16.7) | | 0.79 (0.65-0.95) | | 0.86 (0.79-0.94) | | |
|  | 2013-2016 | 1,363 | 368 | | 18.7% (16.9-20.7) | | 1.00 (0.83-1.19) | | 0.86 (0.80-0.94) | | |
| **Non-diabetes and obstructive CAD (n=19,077)**  *Percutaneous coronary intervention* | | | | | | | | | | |  |
|  | 2004-2006 | 4,530 | 2,511 | | 55.6% (54.2-57.1) | | reference | | reference | | |
|  | 2007-2009 | 5,144 | 2,889 | | 56.4% (55.0-57.7) | | 1.03 (0.96-1.12) | | 1.05 (0.97-1.14) | | |
|  | 2010-2012 | 4,219 | 2,468 | | 58.6% (57.1-60.1) | | 1.14 (1.05-1.24) | | 1.17 (1.08-1.27) | | |
|  | 2013-2016 | 5,184 | 2,996 | | 57.9% (56.5-59.2) | | 1.10 (1.02-1.19) | | 1.12 (1.03-1.21) | | |
| *Coronary artery bypass grafting* | | | | | | | | | | |  |
|  | 2004-2006 | 4,530 | 1,170 | | 17.4% (16.7-21.1) | | reference | | reference | | |
|  | 2007-2009 | 5,144 | 1,191 | | 15.1% (14.2-16.0) | | 0.87 (0.79-0.95) | | 0.85 (0.79-0.94) | | |
|  | 2010-2012 | 4,219 | 989 | | 15.4% (14.5-16.4) | | 0.89 (0.81-0.98) | | 0.88 (0.80-0.97) | | |
|  | 2013-2016 | 5,184 | 1,172 | | 14.7% (13.8-15.5) | | 0.84 (0.77-0.93) | | 0.83 (0.76-0.91) | | |
| *Adjusted for sex, age, smoking, hypertension, previous ischemic stroke, peripheral artery disease, statin treatment, antiplatelet treatment, and oral anti-coagulant treatment. | | | | | | | | | | |  |
